# Glaucomatous Optic Nerve Changes and Thyroid Dysfunction in an Urban South Korean Population

**DOI:** 10.1155/2017/8280209

**Published:** 2017-05-02

**Authors:** Yu Sam Won, Da Yeong Kim, Joon Mo Kim

**Affiliations:** ^1^Department of Neurosurgery, Kangbuk Samsung Hospital, Sungkyunkwan University School of Medicine, Seoul, Republic of Korea; ^2^Department of Ophthalmology, Kangbuk Samsung Hospital, Sungkyunkwan University School of Medicine, Seoul, Republic of Korea

## Abstract

*Purpose.* This study was performed to evaluate the relationship between intraocular pressure (IOP) and glaucomatous optic nerve change and thyroid factors in Korean population. *Materials and Methods.* The study included subjects who underwent health screening in Kangbuk Samsung Hospital. Detailed history taking and systemic and ocular examination including fundus photography were performed for all participants. All fundus photographs were divided into two groups based on disc and RNFL appearance: nonglaucoma and glaucoma group. Subjects were also divided into quartiles of each thyroid function parameter, and the relationship with IOP and glaucoma were analysed. *Results.* In univariate analysis, free T4, T3, and TSH in normal subjects and T3 in thyroid disease group were associated with the IOP. After adjusting for age and sex, the IOP tended to slightly decrease according to the level of the quartile of free T4 and T3 in normal subjects. In terms of glaucoma, on multivariate analysis, it did not show a significant correlation with any thyroid function tests. *Conclusions.* In normal subjects, the IOP tended to be decreased according to the level of free T4 and T3 but the amounts were clinically insignificant. Thyroid factors are not an independent risk factor for the development of glaucoma.

## 1. Introduction

Glaucoma is a chronic optic neuropathy caused by a variety of risk factors and characterized by the progressive loss of retinal ganglion cells (RGC). Intraocular pressure (IOP) is the most important risk factor; however, glaucoma can occur in a low IOP state, and glaucomatous progression can occur during IOP-lowering therapy [1–4]. Therefore, many studies have been conducted to investigate risk factors including IOP and other risk factors [5–7].

Hormones might affect the development of glaucoma and IOP through various mechanisms, and some studies have reported that thyroid hormone affects IOP. Thyroid hormones play a key role in metabolism and homeostasis [8]. Thyroid-associated ophthalmopathy has been described clinically; however, the relationship between thyroid function and glaucoma has remained controversial [9]. Some studies have reported a significant relationship, while others have not. Forte et al. [10] suggested that ocular hypertension (OHT) patients with Graves' orbitopathy showed diffuse abnormalities of the VF and RNFL thinning.

Few studies have been conducted to reveal the relationship between thyroid dysfunction and glaucoma based on the large number of participants. The relationship between thyroid dysfunction and glaucoma is still under debate. In this study, we evaluated the association between IOP, glaucomatous optic nerve change, and thyroid dysfunction.

## 2. Materials and Methods

This study was conducted in two Kangbuk Samsung Hospital Screening Centers in Seoul and Suwon, South Korea, and followed a retrospective, cross-sectional design. This study followed the tenets of the Declaration of Helsinki and was approved by the institutional review board/ethics committee of Kangbuk Samsung Hospital. The study population consisted of individuals who participated in a comprehensive health screening programme at Kangbuk Samsung Hospital, Seoul, Korea. The purpose of this screening programme was to promote health through early detection of chronic diseases and their risk factors. In addition, the Korean Industrial Safety and Health Law requires working individuals to participate in an annual or a biennial health examination. About 60% of the participants were employees of companies or local governmental organizations and their spouses. The remaining participants registered individually for the programme [11]. Subjects who underwent health screening from August 2012 to July 2013 and who were 20 years or older were enrolled in this study. Systemic examinations, including laboratory tests and sociodemographic and behavioural questionnaires, were administered to all subjects, and their medical histories were reviewed to determine the presence of associated systemic disease. Digital fundus images were collected with a digital fundus camera (CR6–45NM; Canon Inc., Tokyo, Japan). IOP was measured twice in each subject using a noncontact tonometer (TX–F; Canon Inc., Tokyo, Japan), and the mean value of two IOP readings was recorded. In cases of both eyes eligible, the right eye from each subject was considered in statistical analysis. Normal subjects were defined as the persons who did not show any glaucoma/glaucoma suspect findings in the fundus and did not have any medical or surgical history to effect on IOP including thyroid orbitopathy. Diabetes is diagnosed as previous diagnosis or treatment history and fasting plasma glucose level ≥ 7.0 mmol/L (126 mg/dL). Hypertension is diagnosed as a systolic blood pressure ≥ 140 mmHg, a diastolic blood pressure ≥ 90 mmHg, or current use of antihypertensive drugs. Hyperlipidaemia is diagnosed as abnormally elevated serum level of any or all lipids and/or lipoproteins. We all excluded glaucoma/glaucoma suspect patients and subjects who underwent glaucoma surgery or laser treatment from normal subjects. In glaucoma participants, data from the eyes with glaucoma were used. All fundus photographs were reviewed by two ophthalmologists, two glaucoma specialists, and one retina specialist who were all blinded to demographic features and laboratory findings. The grading was performed independently by ophthalmologists. Any discrepancies among the observers were resolved by consensus between two glaucoma specialists. First, each fundus photograph was classified into one of the two categories based on disc and RNFL appearance: nonglaucomatous or glaucomatous. A glaucomatous RNFL defect was defined as a localized wedge-shaped RNFL defect within 60 degrees running toward or touching the optic disc border. Nonglaucomatous RNFL defects included superior segmental optic hypoplasia and slit defect. A patient was defined as having a glaucomatous optic neuropathy if the vertical cup-disc ratio (VCDR) was ≥0.7 or if the VCDR asymmetry between the right and left eyes was ≥0.2. In cases with newly detected glaucomatous features, glaucoma evaluations were recommended and some of them were determined to have glaucoma or not after undergoing glaucoma evaluation at the hospital. Finally, subjects who had not been previously diagnosed with glaucoma or had not visited a hospital for glaucoma evaluation were placed into either category 2 or 3, according to the criteria set forth by the International Society of Geographical and Epidemiological Ophthalmology (ISGEO) [12]. Category 1 requires a VF defect consistent with glaucoma and either a vertical cup-disc ratio (VCDR) ≥ 0.7 (97.5th percentile) or ≥0.2 VCDR asymmetry between the right and left eyes (97.5th percentile). Category 2 indicates that the VF results are not definitive, requiring a VCDR ≥ 0.9 (99.5th percentile) or VCDR asymmetry ≥ 0.3 (99.5th percentile). Category 3 indicates that there are no data available regarding VF testing or optic disc examination; therefore, visual acuity < 3/60 and an IOP greater than the 99.5th percentile for this population (IOP = 21 mmHg) are required.

Optic disc haemorrhage was defined as an isolated haemorrhage on or around the optic disc or in the peripapillary retina within 1-disc diameter. Subjects with alternative causes of retinal haemorrhage, such as ischemic optic neuropathy, papillitis, retinal vein occlusion, diabetic retinopathy, or posterior vitreous detachment, were excluded. We also excluded participants with low-quality fundus photographs due to media opacity or a small pupil in order to discriminate glaucoma patients, as well as patients who underwent glaucoma surgery and laser treatment and who were on systemic medications that could affect IOP, such as a *β*-blocker.

Subjects were divided equally into four groups (quartiles) based on thyroid laboratory testing as follows: thyroid-stimulating hormone (TSH, *μ*IU/mL) quartiles, Q1 (TSH < 1.30), Q2 (1.30 ≤ TSH < 1.90), Q3 (1.90 ≤ TSH < 2.75), and Q4 (TSH ≥ 2.75); free thyroxine (free T4, ng/dL) quartiles, Q1 (free T4 < 1.15), Q2 (1.15 ≤ free T4 < 1.26), Q3 (1.26 ≤ free T4 < 1.38), and Q4 (free T4 ≥ 1.38); and triiodothyronine (T3, pg/mL) quartiles, Q1 (T3 < 2.87), Q2 (2.87 ≤ T3 < 3.12), Q3 (3.12 ≤ T3 < 3.40), and Q4 (T3 ≥ 3.40). This dividing analysis method was used to it at previous study with blood chemistry data [13]. History of thyroid abnormalities and newly diagnosed subjects were classified into hypothyroidism, hyperthyroidism, thyroid nodular cyst, and thyroid hormone treatment.

All statistical analyses were performed with PASW Statistics 18.0 (IBM, Armonk, New York, USA). Each variable was initially evaluated using the chi-square test, and significant variables (*p* < 0.1) were included in multivariate logistic regression. Multivariate logistic regression with stepwise selection while controlling for all confounding variables (*p* < 0.1) was used to evaluate the independent risk factors associated with glaucoma. Odds ratios (OR) with 95% confidence intervals (CI) were estimated using logistic regression models. In all cases, a *p* < 0.05 was considered statistically significant.

## 3. Results

Of 168,044 patients treated at the two screening centers, 164,029 (97.61%) were enrolled in this study. The remaining 4015 subjects were excluded because fundus photographs could not be obtained or because low-quality photographs could not be reliably evaluated due to media opacity or small pupil. Of the 164,029 participants, 93,559 (57.0%) were male and 70,470 (43.0%) were female. The mean age was 40.56 ± 8.51 years. Optic disc haemorrhage was noted in 226 (0.14%) subjects. Of the total 164,029 participants, glaucomatous fundus photographs were obtained in 2454 (1.50%) subjects (apart from the ISGEO criteria, according to the results of optic disc and RNFL appearance in fundus photographs). 1477 subjects (60.2%) of 2454 had been previously diagnosed with glaucoma. Of the remaining 977 subjects, 313 subjects underwent glaucoma evaluations including red-free fundus photography, optical coherence tomography, and visual field testing in our glaucoma clinic. Among these 313 subjects, 162 and 128 were diagnosed as having definite and suspected glaucoma (early glaucoma with RNFL defect), respectively, whereas the remaining 23 did not have glaucoma. The remaining 664 subjects were considered to have glaucoma according to the ISGEO criteria and/or presence of RNFL defect. Glaucoma evaluation including visual field test was not available in 664 subjects; therefore, subjects were diagnosed as ISGEO category 3 (visual acuity < 3/60 and an IOP greater than the 99.5th percentile for this population are required). All eligible cases for glaucoma group showed RNFL defect. The demographic data are shown in Table 1.

On univariate analysis, there was a significant difference in T3 level (*p* < 0.001, chi-square test) between patients with and without glaucomatous optic neuropathy (Table 2). There was no relationship between disc haemorrhage and thyroid function on univariate analysis. The relationship between thyroid function and IOP was shown in Tables 3 and 4. On univariate analysis of normal population, as the quartile of free T4 and T3 increased, the IOP tended to increase slightly. On multivariate analysis adjusted for age, sex, and comorbidities (diabetes, hypertension, hyperlipidaemia, and current smoker), however, the IOP in the first quartile of free T4 and T3 were higher than the second or other quartiles significantly (Table 5). In analysis of the odds ratio between thyroid dysfunction and glaucoma, thyroid function was not associated with glaucoma (Tables 6 and 7).

## 4. Discussion

Thyroid hormone affects the maintenance of homeostasis in the body and regulates the basal body metabolism [8]. Many studies have tried to determine the association between hypothyroidism and glaucoma. In our study, we did not find that thyroid dysfunction was an independent risk factor of glaucoma development. On univariate analysis, the presence of glaucomatous optic neuropathy was significantly influenced by the level of T3, but the association did not remain statistically significant on multivariate regression. Disc haemorrhage was not associated with thyroid dysfunction as well.

Considering previous reports about an association between IOP and thyroid function, there was a possible theoretical relationship between thyroid function and glaucoma. Hyperthyroidism might induce IOP increase through increased intraorbital pressure or contraction of enlarged extraocular muscles [14, 15]. The main mechanism could be elevation of episcleral venous pressure secondary to increasing of intraorbital content and pressure. The increased IOP in hypothyroidism could be due to the excessive accumulation of mucopolysaccharides in the trabecular meshwork [16, 17]. Smith et al. [16] reported that the prevalence of hypothyroidism in the primary open-angle glaucoma group was higher than those in the control subjects in their case-control study. They proposed that, in the untreated hypothyroid state, hyaluronic acid accumulates excessively in the trabecular meshwork and/or aqueous, causing an obstruction to facility of outflow. This phenomenon was thought to be due to decreased enzyme activity in the hypothyroid state and decreased degradation of hyaluronic acid. After treatment of hypothyroidism, the outflow of aqueous humor was recovered. Some studies have reported that treating hypothyroidism decreases IOP through increasing aqueous outflow. Centanni et al. [17] reported that the IOP of subjects who showed a significantly higher IOP than the control group of subclinical hypothyroidism were decreased approximately by 3 mmHg after treatment of hypothyroidism. Bahceci et al. [18] reported that hypothyroidism reversibly induced increasing IOP. On the other hand, Cheng and Perkins [19] did not find a statistically significant difference in the distribution of IOP between hypothyroidism and normal control groups. However, their study included thyroid hormone-treated patients, which could have affected the results due to the reversible effects of the treatment. McLenachan and Davies [20] reported that high IOP was associated with hypothyroidism due to changes in the quantity and quality of mucopolysaccharides in the trabecular meshwork.

Physiologically, TSH level and T3 and T4 levels are adjusted in relation to each other [8]. In the hyperthyroid state, blood tests typically show low thyroid-stimulating hormone (TSH) and elevated triiodothyronine (T3) and thyroxine (T4) levels. Otherwise, an elevated TSH indicates that the thyroid gland is not producing enough thyroid hormone (hypothyroid state). In our study, the IOP in the first quartile of free T4 and T3 were higher than those in the second or other quartiles. These results were statistically significant, but the changes were clinically insignificant. These results can be considered the several reasons that might appear. First, this study included a very large number of subjects and the results for each group might be distributed evenly. Due to very large number of the enrolled subjects in the study, statistically significant result was showed. However, the difference was very small and might not be noticeable. Second, in normal population, the structural abnormalities of the extraocular muscles or intraorbital tissues that could affect the IOP were less likely to appear. Therefore, the difference of IOP between groups was less likely to emerge.

Many studies have investigated the association between glaucoma and thyroid dysfunction. In the study of Smith et al. [16], 64 subjects with POAG and 64 controls were evaluated for hypothyroidism. About 23.4% of patients with POAG and 4.7% of controls had hypothyroidism, and the difference was statistically significant. Additionally, in POAG patients, 10.9% showed newly diagnosed hypothyroidism. Munoz-Negrete et al. [21] investigated the prevalence of hypothyroidism in 75 OAG patients and 75 controls. Although applying similar diagnostic criteria as Smith et al. [16], they did not find a statistically significant difference. Gillow et al. [22] investigated this relationship with 100 glaucoma patients using TSH level. Clinical hypothyroidism was present in 4% of participants, and subclinical hypothyroidism was present in 3% of participants. In this study, POAG patients did not show an association with hypothyroidism. Some previous studies about the association between glaucoma and thyroid function were based on their glaucoma diagnosis mainly on IOP, not optic disc evaluation [19, 20, 23, 24].

In the Namil study of Korean subjects, patients with a history of thyroid disease had a 7.4-fold higher risk of open-angle glaucoma than the control group [25]. In the Blue Mountain Eye Study, the incidence of glaucoma was 2.1-fold higher in the thyroxine treatment group than in the control group [26]. Cross et al. [27] reported that the incidence of glaucoma in the thyroid disease group was 1.4-fold higher than in the control group. Lin et al. [28] reported that the hypothyroidism group had a 1.9-fold higher incidence of glaucoma than the control group. Girkin et al. [29] showed significantly higher relative risk of glaucoma in the hypothyroidism group of male subjects that the average age of participants was 69 years.

On the other hand, Karadimas et al. reported a different result. In 100 patients of newly diagnosed hypothyroidism patients, there were no participants with glaucomatous optic nerve damage [30]. Motsko and Jones [31] investigated the risk of OAG with newly diagnosed OAG patients without hypothyroidism. Adjusted for other risk factors, they reported that OAG did not show significant association with hypothyroidism. Haefliger et al. [32] said that glaucoma prevalence does not seem to be significantly increased in thyroid eye disease. Kakigi et al. [33] reported a similar result with us. In this study, participants of National Health Interview Survey were relatively young age (no hypothyroidism; 57.0 ± 12.5, hypothyroidism; 61.5 ± 13.3). The unadjusted analysis showed a significant association between self-reported glaucoma and self-reported hypothyroidism. However, multivariate logistic regression analysis adjusted for age, gender, race, comorbidities, and health-related behavior showed no association between self-reported glaucoma and hypothyroidism or thyroid disease. This study was based on two US nationwide surveys and failed to confirm previously reported associations between hypothyroidism and glaucoma.

In some studies for racial characteristics of thyroid eye disease, the effects of thyroid factor on the eyes were different between races [34, 35]. In the study of the effect of smoking, the results showed tendency of higher prevalence of current smokers in subjects with larger orbital muscle volume in normal population [36]. Thyroid hormone might also have an effect on the extraocular muscles [14, 15]. The prevalence of thyroid eye disease between European and Asian populations and the overall risk for Europeans for developing thyroid eye disease were 6.4 times higher than for Asians [34]. The prevalence was 42% in Europeans compared to 7.7% in Asians [34]. Bednarczuk et al. [35] reported that smoking was a risk factor for thyroid eye disease in Polish people but not in Japanese people. There are reports as described above for the extraocular muscles; however, further studies are necessary to support our results. In our study, the percentage of current smoker did not show a significant difference between glaucoma and normal group.

In this study, glaucomatous optic neuropathy did not show a significant correlation with any thyroid factors. The age may be one of the causes of such results. In our study, the mean age of participants was 40.56 years. Other studies that reported the significant associations between glaucoma and thyroid factors had relatively older participants than in our study; the average age of participants was about 63 to 70 years for each study. The old age may have longer duration of thyroid disease than the young age, and thyroid factor may affect to the eye for a longer period of time in the old age. In addition to this, the number of enrolled subjects (164,029) in this study is a very huge data compared to other studies which have been conducted so far. This factor is thought to be able to level the results of statistics between the groups.

There are some limitations in the present study. The number 2431 that finally included in the glaucoma group is the remainder of 2454 which excluded the 23 subjects of nonglaucoma, finally confirmed. Therefore, glaucoma suspect might be included in the glaucoma group. The difference in prevalence of current smokers might influence the results. This study is not a population-based study. Also, patient selection based on participation in serial health screening might be biased toward higher economic and educational levels. Patients who worry about their health undergo medical examinations more frequently and, therefore, tend to be healthier than those who do not undergo frequent examinations. Our study was a cross-sectional study; however, considering the fact that glaucoma is a progressive disease, a longitudinal approach would also provide a valuable data. In addition, we did not perform glaucoma evaluation on all subjects, so it is difficult to fully evaluate the relationships between ophthalmic factors and other general factors. The use of noncontact tonometry rather than applanation tonometry may also be another limitation. Despite these limitations, this study was performed with a large number of participants and targeted a single race. Our data may be helpful to understand the pathophysiology of glaucoma.

In conclusion, our results showed that the IOP tend to be decreased slightly as the quartile of free T4 and T3 increased. However, the clinical significance may be limited. Our study also suggested that thyroid dysfunction was not an independent risk factor of glaucomatous optic neuropathy. These results may increase the understanding of risk factors of glaucoma. Further prospective study is needed to validate the results.

## Figures and Tables

**Table 1:** Demographic and general health characteristics.

Characteristics	Glaucoma (+) (*n* = 2431)	Glaucoma (−) (*n* = 161,598)	*p* value

Age, years (mean ± SD)	44.10 ± 9.17	40.50 ± 8.50	<0.001^∗^
Male (%)	1736 (71.4%)	91,823 (56.8%)	<0.001^∗∗^
IOP (mmHg)	15.8 ± 3.1	15.0 ± 3.0	<0.001^∗^
Diabetes (%)	157 (6.5)	4732 (2.9)	<0.001^∗∗^
Hypertension (%)	497 (20.4)	13,998 (8.7)	<0.001^∗∗^
Hyperlipidaemia (%)	473 (19.5)	22,332 (13.8)	<0.001^∗∗^
Current smoker (%)	547 (22.5)	33,158 (20.5)	0.033^∗∗^
TSH (*μ*IU/mL)	2.39 ± 3.64	2.26 ± 2.67	0.089^∗^
Free T4 (ng/dL)	1.27 ± 0.25	1.26 ± 0.23	0.080^∗^
T3 (pg/mL)	3.21 ± 0.84	3.162 ± 0.65	<0.001^∗^
Hyperthyroidism (%)	37 (1.5%)	2752 (1.7%)	<0.001^∗∗^
Hypothyroidism (%)	36 (1.5%)	3086 (1.9%)	<0.001^∗∗^
Nodular cyst (%)	138 (5.6%)	11,239 (6.7%)	<0.001^∗∗^
Hormone treatment (%)	48 (2.0%)	3081 (1.8%)	<0.001^∗∗^

Characteristic	Disc haemorrhage (+) (*n* = 226)	Disc haemorrhage (−) (*n* = 163,803)	*p* value

Age, years (mean ± SD)	44.68 ± 8.97	40.55 ± 8.51	<0.001^∗^
Male (%)	151 (66.81%)	93,408 (57.02%)	0.003^∗∗^

Continuous values are presented as mean ± standard deviation (SD).

Categorical values are presented as percentage (%).

^∗^Student's *t-*test, ^∗∗^Chi-square test.

**Table 2:** Univariate analysis of glaucoma and thyroid function.

	Quartiles of thyroid hormone				*p* value
	1st quartile	2nd quartile	3rd quartile	4th quartile	
Glaucoma (+) (*n* = 2431)					
TSH (*μ*IU/mL) (*n* = 2449) (0.01 ~ 100)	635 (25.93%)	612 (24.99%)	604 (24.66%)	598 (24.42%)	0.943^∗^
Free T4 (ng/dL) (*n* = 2442) (0.06 ~ 7.70)	583 (23.87%)	638 (26.13%)	625 (25.59%)	596 (24.41%)	0.090^∗^
T3 (pg/mL) (*n* = 2077) (0.34 ~ 32.60)	438 (21.09%)	461 (22.20%)	612 (29.47%)	566 (27.25%)	<0.001^∗^

^∗^Chi-square test

TSH: 1st quartile < 1.30, 1.30 ≤ 2nd quartile < 1.90, 1.90 ≤ 3rd quartile < 2.75, 4th quartile ≥ 2.75; free T4: 1st quartile < 1.15, 1.15 ≤ 2nd quartile < 1.26, 1.26 ≤ 3rd quartile < 1.38, 4th quartile ≥ 1.38; T3: 1st quartile < 2.87, 2.87 ≤ 2nd quartile < 3.12, 3.12 ≤ 3rd quartile < 3.40, 4th quartile ≥ 3.40.

**Table 3:** Univariate analysis of thyroid function and IOP in a normal population without glaucoma.

Variables	IOP (mmHg)		*p* value
TSH (*μ*IU/mL)			<0.001^∗^
1st quartile (<1.30) (*n* = 39,894) (25.91%)	14.94 ± 2.96		
2nd quartile (1.30–1.90) (*n* = 39,402) (25.58%)	15.02 ± 2.96		
3rd quartile (1.90–2.75) (*n* = 38,013) (24.68%)	15.04 ± 2.94		
4th quartile (≥2.75) (*n* = 36,681) (23.83%)	15.02 ± 2.92		

Free T4 (ng/dL)			<0.001^∗^
1st quartile (<1.15) (*n* = 39,071) (25.43%)	14.92 ± 2.94		
2nd quartile (1.15–1.26) (*n* = 39,972) (26.01%)	15.01 ± 2.95		
3rd quartile (1.26–1.38) (*n* = 39,268) (25.55%)	15.05 ± 2.95		
4th quartile (≥1.38) (*n* = 35,350) (23.01%)	15.05 ± 2.95		

T3 (pg/mL)			<0.001^∗^
1st quartile (<2.87) (*n* = 33,064) (24.36%)	14.64 ± 2.95		
2nd quartile (2.87–3.12) (*n* = 31,796) (23.44%)	14.88 ± 2.95		
3rd quartile (3.12–3.40) (*n* = 35,356) (26.06%)	15.10 ± 2.96		
4th quartile (≥3.40) (*n* = 35,466) (26.14%)	15.19 ± 2.96		

Characteristics of disease	(+)	(−)	*p* value

Hypothyroidism	14.65 ± 2.93	15.01 ± 2.95	<0.001^∗∗^
Hyperthyroidism	14.87 ± 2.92	15.01 ± 2.95	0.017^∗∗^
Nodular cyst	14.92 ± 2.90	15.01 ± 2.95	0.003^∗∗^
Hormone treatment	14.82 ± 2.93	15.01 ± 2.95	0.001^∗∗^

Continuous values are presented as mean ± standard deviation (SD).

^∗^One-way ANOVA, ^∗∗^Student's *t*-test.

**Table 4:** Univariate analysis of thyroid function and IOP in thyroid disease patients without glaucoma.

Variables	IOP (mmHg)		*p* value
TSH (*μ*IU/mL)			0.128^∗^
1st quartile (<1.30) (*n* = 3677) (32.32%)	14.79 ± 2.92		
2nd quartile (1.30–1.90) (*n* = 1788) (15.72%)	14.80 ± 2.80		
3rd quartile (1.90–2.75) (*n* = 2020) (17.75%)	14.68 ± 2.85		
4th quartile (≥2.75) (*n* = 3891) (34.21%)	14.87 ± 2.91		

Free T4 (ng/dL)			0.762^∗^
1st quartile (<1.15) (*n* = 3755) (33.12%)	14.76 ± 2.89		
2nd quartile (1.15–1.26) (*n* = 2581) (22.76%)	14.81 ± 2.85		
3rd quartile (1.26–1.38) (*n* = 2101) (18.53%)	14.81 ± 2.88		
4th quartile (≥1.38) (*n* = 2901) (25.59%)	14.84 ± 2.92		

T3 (pg/mL)			<0.001^∗^
1st quartile (<2.87) (*n* = 4171) (41.69%)	14.60 ± 2.92		
2nd quartile (2.87–3.12) (*n* = 2292) (22.91%)	14.77 ± 2.86		
3rd quartile (3.12–3.40) (*n* = 1818) (18.17%)	14.95 ± 2.88		
4th quartile (≥3.40) (*n* = 1724) (17.23%)	14.90 ± 2.91		

Characteristics of disease	(+)	(−)	*p* value

Hypothyroidism	14.65 ± 2.90	14.85 ± 2.88	0.001^∗∗^
Hyperthyroidism	14.87 ± 2.94	14.78 ± 2.87	0.161^∗∗^
Nodular cyst	14.88 ± 2.86	14.74 ± 2.91	0.016^∗∗^
Hormone treatment	14.86 ± 2.95	14.78 ± 2.87	0.191^∗∗^

Continuous values are presented as mean ± standard deviation (SD).

^∗^One-way ANOVA, ^∗∗^Student's *t*-test.

**Table 5:** Adjusted coefficients and 95% confidence intervals of IOP stratified by each thyroid function parameter in a normal population without glaucoma.

Variables	Coefficients	95% confidence interval	*p* value^∗^
Age	0.025	(0.024–0.027)	<0.001
Sex (male)	0.726	(0.693–0.759)	<0.001
Diabetes	0.697	(0.609–0.784)	<0.001
Hypertension	0.381	(0.326–0.436)	<0.001
Hyperlipidaemia	0.150	(0.106–0.194)	<0.001
Current smoker	0.234	(0.194–0.275)	<0.001
TSH (*μ*IU/mL)			
1st quartile (<1.30)	0 (reference)		
2nd quartile (1.30–1.90)	0.074	(0.033–0.115)	<0.001
3rd quartile (1.90–2.75)	0.135	(0.094–0.177)	<0.001
4th quartile (≥2.75)	0.159	(0.117–0.200)	<0.001

Age	0.025	(0.023–0.026)	<0.001
Sex (male)	0.766	(0.732–0.801)	<0.001
Diabetes	0.702	(0.614–0.789)	<0.001
Hypertension	0.384	(0.329–0.439)	<0.001
Hyperlipidaemia	0.146	(0.102–0.190)	<0.001
Current smoker	0.220	(0.180–0.259)	<0.001
Free T4 (ng/dL)			
1st quartile (<1.15)	0 (reference)		
2nd quartile (1.15–1.26)	−0.025	(−0.066–0.016)	0.239
3rd quartile (1.26–1.38)	−0.087	(−0.129 to −0.044)	<0.001
4th quartile (≥1.38)	−0.176	(−0.221 to −0.131)	<0.001

Age	0.025	(0.023–0.027)	<0.001
Sex (male)	0.737	(0.702–0.773)	<0.001
Diabetes	0.693	(0.606–0.781)	<0.001
Hypertension	0.381	(0.326–0.436)	<0.001
Hyperlipidaemia	0.152	(0.108–0.196)	<0.001
Current smoker	0.216	(0.176–0.256)	<0.001
T3 (pg/mL)			
1st quartile (<2.87)	0 (reference)		
2nd quartile (2.87–3.12)	−0.102	(−0.143 to −0.060)	<0.001
3rd quartile (3.12–3.40)	−0.043	(−0.085 to −0.001)	0.045
4th quartile (≥1.38)	−0.050	(−0.094 to −0.006)	0.027

Hypothyroidism	−0.091	(−0.200–0.017)	0.099
Hyperthyroidism	0.042	(−0.071–0.156)	0.465
Nodular cyst	0.071	(0.011–0.130)	0.019
Hormone treatment	−0.004	(−0.113–0.104)	0.938

Adjusted for age, sex, diabetes, hypertension, hyperlipidaemia, and current smoker.

Diabetes is diagnosed as previous diagnosis or treatment history of diabetes or fasting plasma glucose level ≥ 7.0 mmol/L (126 mg/dL).

Hypertension is diagnosed as a systolic blood pressure ≥ 140 mmHg, a diastolic blood pressure ≥ 90 mmHg, or current use of antihypertensive drugs.

Hyperlipidaemia is diagnosed as abnormally elevated serum level of any or all lipids and/or lipoproteins.

^∗^Multivariate linear regression analysis.

**Table 6:** Adjusted odds ratio and 95% confidence intervals of glaucoma stratified by thyroid function.

Variables	Odds ratio	95% confidence interval	*p* value^∗^
Age	1.029	(1.024–1.035)	<0.001
Sex (male)	1.783	(1.583–2.007)	<0.001
Diabetes	1.087	(0.878–1.345)	0.445
Hypertension	1.860	(1.632–21,192)	<0.001
Hyperlipidaemia	0.963	(0.850–1.090)	0.547
Current smoker	0.878	(0.781–0.988)	0.031

T3 (pg/mL)	(All subjects)		
1st quartile (<2.87)	1 (reference)		
2nd quartile (2.87–3.12)	1.008	(0.873–1.164)	0.915
3rd quartile (3.12–3.40)	1.134	(0.984–1.306)	0.082
4th quartile (≥3.40)	1.036	(0.892–1.203)	0.646

T3 (pg/mL)	(20 ~ 39 age subjects)		
1st quartile (<2.87)	1 (reference)		
2nd quartile (2.87–3.12)	0.912	(0.697–1.193)	0.500
3rd quartile (3.12–3.40)	1.065	(0.822–1.380)	0.634
4th quartile (≥3.40)	0.966	(0.740–1.260)	0.797

T3 (pg/mL)	(40 ~ 59 age subjects)		
1st quartile (<2.87)	1 (reference)		
2nd quartile (2.87–3.12)	1.071	(0.895–1.282)	0.452
3rd quartile (3.12–3.40)	1.153	(0.962–1.381)	0.123
4th quartile (≥3.40)	1.056	(0.871–1.280)	0.581

T3 (pg/mL)	(60 or older age subjects)		
1st quartile (<2.87)	1 (reference)		
2nd quartile (2.87–3.12)	0.823	(0.467–1.450)	0.500
3rd quartile (3.12–3.40)	1.200	(0.695–2.070)	0.514
4th quartile (≥3.40)	1.086	(0.565–2.087)	0.804

Adjusted for age, sex, diabetes, hypertension, hyperlipidaemia, and current smoker.

Characteristics of disease (hypothyroidism, hyperthyroidism, nodular cyst, hormone treatment) include both medical history review and currently diagnosed disease.

Diabetes is diagnosed as fasting plasma glucose level ≥ 7.0 mmol/L (126 mg/dL).

Hypertension is diagnosed as a systolic blood pressure ≥ 140 mmHg, a diastolic blood pressure ≥ 90 mmHg, or current use of antihypertensive drugs.

Hyperlipidaemia is diagnosed as abnormally elevated serum level of any or all lipids and/or lipoproteins.

^∗^Multivariate logistic regression analysis.

**Table 7:** The odds ratio between thyroid factors and glaucoma.

Variables	Glaucoma (+)	(−)	Odds ratio	95% CI	*p* ^∗^
Hyperthyroidism			0.905	(0.653–1.255)	0.550
(+) (*n* = 2789)	37	2752			
(−) (*n* = 163,361)	2391	160,970			

Hypothyroidism			0.783	(0.563–1.091)	0.147
(+) (*n* = 3122)	36	3086			
(−) (*n* = 163,023)	2392	160,631			

Nodular cyst			0.817	(0.688–0.972)	0.022
(+) (*n* = 11,377)	138	11,239			
(+) (*n* = 154,949)	2293	152,656			

Hormone treatment			1.052	(0.789–1.403)	0.730
(+) (*n* = 3129)	48	3081			
(+) (*n* = 163,079)	2380	160,699			

^∗^Chi-square test.
